# Glucocorticoids Impair Phagocytosis and Inflammatory Response Against Crohn’s Disease-Associated Adherent-Invasive *Escherichia coli*

**DOI:** 10.3389/fimmu.2018.01026

**Published:** 2018-05-16

**Authors:** Mauricio Javier Olivares-Morales, Marjorie Katherine De La Fuente, Karen Dubois-Camacho, Daniela Parada, David Diaz-Jiménez, Alejandro Torres-Riquelme, Xiaojiang Xu, Nayaret Chamorro-Veloso, Rodrigo Naves, Maria-Julieta Gonzalez, Rodrigo Quera, Carolina Figueroa, John Anthony Cidlowski, Roberto Mauricio Vidal, Marcela Alejandra Hermoso

**Affiliations:** ^1^Innate Immunity Laboratory, Immunology Program, Faculty of Medicine, Biomedical Sciences Institute, Universidad de Chile, Santiago, Chile; ^2^Laboratory of Integrative Bioinformatics, Department of Health and Human Services, National Institute of Environmental Health Sciences, National Institutes of Health, Durham, NC, United States; ^3^Enteropathogens Laboratory, Microbiology and Mycology Program, Faculty of Medicine, Biomedical Sciences Institute, Universidad de Chile, Santiago, Chile; ^4^Neuroimmunology Laboratory, Immunology Program, Faculty of Medicine, Biomedical Sciences Institute, Universidad de Chile, Santiago, Chile; ^5^Cell Biology Program, Faculty of Medicine, Biomedical Sciences Institute, Universidad de Chile, Santiago, Chile; ^6^Gastroenterology Department, Clínica Las Condes, Santiago, Chile; ^7^Signal Transduction Laboratory, Department of Health and Human Services, National Institute of Environmental Health Sciences, National Institutes of Health, Durham, NC, United States

**Keywords:** glucocorticoids, macrophages, adherent-invasive *Escherichia coli*, phagocytosis, inflammatory response, Crohn’s disease

## Abstract

Crohn’s disease (CD) is a chronic inflammatory bowel disorder characterized by deregulated inflammation triggered by environmental factors. Notably, adherent-invasive *Escherichia coli* (AIEC), a bacterium with the ability to survive within macrophages is believed to be one of such factors. Glucocorticoids are the first line treatment for CD and to date, it is unknown how they affect bactericidal and inflammatory properties of macrophages against AIEC. The aim of this study was to evaluate the impact of glucocorticoid treatment on AIEC infected macrophages. First, THP-1 cell-derived macrophages were infected with a CD2-a AIEC strain, in the presence or absence of the glucocorticoid dexamethasone (Dex) and mRNA microarray analysis was performed. Differentially expressed mRNAs were confirmed by TaqMan-qPCR. In addition, an amikacin protection assay was used to evaluate the phagocytic and bactericidal activity of Dex-treated macrophages infected with *E. coli* strains (CD2-a, HM605, NRG857c, and HB101). Finally, cytokine secretion and the inflammatory phenotype of macrophages were evaluated by ELISA and flow cytometry, respectively. The microarray analysis showed that CD2-a, Dex, and CD2-a + Dex-treated macrophages have differential inflammatory gene profiles. Also, canonical pathway analysis revealed decreased phagocytosis signaling by Dex and anti-inflammatory polarization on CD2-a + Dex macrophages. Moreover, amikacin protection assay showed reduced phagocytosis upon Dex treatment and TaqMan-qPCR confirmed Dex inhibition of three phagocytosis-associated genes. All bacteria strains induced TNF-α, IL-6, IL-23, CD40, and CD80, which was inhibited by Dex. Thus, our data demonstrate that glucocorticoids impair phagocytosis and induce anti-inflammatory polarization after AIEC infection, possibly contributing to the survival of AIEC in infected CD patients.

## Introduction

Crohn’s disease (CD) is a chronic inflammatory disorder of the gastrointestinal tract. Principal signs and symptoms of CD include diarrhea, intestinal bleeding, abdominal pain, anemia, and weight loss; with complications, such as fistulas, abscesses, granulomas, and fibrosis, leading to surgical resection of compromised intestinal tissue (1). It is a multifactorial disease with genetic polymorphisms and associated environmental factors (1), one such being adherent-invasive *Escherichia coli* (AIEC) strains, which have received increased research focus in recent years.

Adherent-invasive *Escherichia coli* are a heterogeneous group of *E. coli* bacteria that are so named for their ability to adhere and to invade epithelial cells. They have increased survival ability within macrophages, where they induce continuous inflammatory cytokine secretion *in vitro* (2–4), possibly being a key event triggering inflammation and affecting CD chronicity (5). In addition, macrophages are found in greater numbers on inflamed tissue in CD patients (6). Some AIEC strains have been sequenced and well described and are therefore used as reference strains. For instance, strain HM605 (7) has been studied in susceptibility to antibiotics (8) and CD etiology (9), while NRG857c has also been studied in regards to virulence factors and iron acquisition (10).

We previously described *E. coli* strains that have AIEC features, specifically CD2-a *E. coli* strain, which has high survival rates within macrophages compared to other *E. coli* with AIEC feature or NRG857c (11). Although reference AIEC strains belong to the same pathogen group, they differ greatly in their genome sequence and virulence gene composition. An example being LF82 closely related to NRG857c (10), while HM605 and CD2-a *E. coli* strain are further apart from them (11). To date, it is unknown whether different strains have altering effects on the immune response.

One of the most important and well-studied predisposing risk factors related to CD is NOD2 polymorphisms since 30–40% of patients hold this modification (12). NOD2 is a cytoplasmic receptor belonging to the group of pattern recognition receptors (PRRs) that recognizes muramyl dipeptide (a component of the bacterial cell wall). Abundant in immune system cells such as macrophages and epithelial cells, its expression can be induced by inflammatory cytokines such as TNF-α and IFN-γ. NOD2 leads to NF-κB transcription factor activation resulting in pro-inflammatory cytokine production. NOD2 polymorphisms can result in gain or loss of function and both have been associated with CD (13).

Macrophages are key participants in the immune system, residing in all tissues and having different functions in each. Their main role is to remove and initiate inflammatory responses against pathogens they recognize through activation of PRRs (14). In addition, pathogens can be recognized through activation of scavenger receptors (14). Phagocytosis by macrophages can be triggered by activation of multiple receptors and PRRs, such as FcγR (15), CR1 (16), CD48 (17), and CD14 (18), among others. In time, phagosomes mature increasing their bactericidal mechanisms, such as an acidic pH, production of reactive oxygen species and nitric oxide [by the action of the Nox2 and inducible nitric oxide synthase (iNOS) enzymes], and upregulation of proteolytic enzymes (19). Simultaneously, upregulation of CD40, MHC class II, and CD80 takes place, which are necessary in order to present antigens to CD4+ T lymphocytes (20). Macrophages also produce inflammatory cytokines, such as TNF-α and IL-23, that direct CD4+ T lymphocyte responses to Th1 and Th17 (21). Historically, this inflammatory macrophage phenotype has been called M1 and works in opposition to the anti-inflammatory M2 phenotype (22). Polarization to M1 and M2 phenotypes is achieved by the action of inflammatory Th1 cytokines together with pathogen antigens, and Th2 cytokines or anti-inflammatory stimuli such as IL-10 or glucocorticoids, respectively (21, 22).

Glucocorticoids are hormones secreted by the adrenal glands in response to stress and its receptor is located in all tissues affecting the cardiovascular, developmental, reproductive, and immune systems (23). Also, glucocorticoids have a well-described anti-inflammatory effect, such as cytokine secretion inhibition, neutrophil extravasation impairment, and induction of immune cell apoptosis. Although recent findings have shown that they can also induce pro-inflammatory changes (23, 24).

Glucocorticoids polarize macrophages toward an M2-like phenotype, resulting in increased expression of anti-inflammatory cytokines and phagocytic activity against apoptotic cells (24), which are used to treat inflammatory disorders (25). In the USA, they are used in approximately 1.2% of the population (26), while in the UK their rate is estimated to be 0.9% (25). Treatment of CD patients with glucocorticoids resulted in a total disappearance of symptoms in 58% of cases, while 26% experienced a partial reduction in symptoms and 16% no response at all (27). It is accepted that glucocorticoid high dosage can increase the risk of bacterial infection in rheumatoid arthritis (28), chronic obstructive pulmonary disease (29), or lupus (28). A study addressing the causes of bacterial co-infection with H1N1 influenza showed that glucocorticoid release from the adrenal glands was necessary to impair the immune system, allowing bacterial growth in the organism (30).

So far, it remains unknown how glucocorticoids affect the interaction between macrophages and AIEC, or whether glucocorticoids even help bacterial survival, as studies on their bactericidal effects are scarce. One study from the 1980’s showed that they can decrease the bactericidal activity of macrophages against bacteria (31), although several contradictory results showed increased (32, 33) or decreased (29, 34) phagocytosis. Moreover, NOD2 polymorphisms have been described to alter neutrophil and monocytes bactericidal effect, with one polymorphism increasing phagocytosis (35) while NOD2 loss of function decreases phagocytosis (36). At present, it is unknown whether glucocorticoids can modulate phagocytosis in patients where it is already impaired by genetic predisposition.

Here, we seek to provide new insights on the interaction between macrophages and AIEC, and the effect of glucocorticoid treatment from a bactericidal and inflammatory perspective, by using the AIEC strain CD2-a and PMA-activated THP-1 cell line macrophages concurrent with bone marrow-derived macrophages (BMDMs) from *Nod2*-knockout (*Nod2−/−*) mice as CD macrophage model.

## Materials and Methods

### Cell Line

A human THP-1 cell line (ATCC^®^ TIB-202™) was maintained in RPMI 1640 (Corning) supplemented with 10% fetal bovine serum (FBS) (LifeTechnologies), 5 µM β-mercaptoethanol (Sigma), 100 U/mL penicillin, and 100 µg/mL streptomycin and maintained at 37°C and 5% CO_2_. For macrophage differentiation, a 72-h protocol was standardized in our laboratory. Briefly, cells were treated with 10 ng/mL 13-phorbol 12-myristate acetate (PMA) (Sigma) for 48 h, followed by 24 h without the stimuli. THP-1 macrophage phenotype features are shown in Figure S1 in Supplementary Material. BMDMs from *Nod2−/−* and wild-type (WT) mice were obtained as previously described (11, 37). Immortalized BMDMs and Caco-2 cells were maintained in DMEM (Gibco) supplemented with 10% FBS, 100 U/ml penicillin, and 100 µg/mL streptomycin (complete DMEM), plated and cultured at 37°C and 5% CO_2_. For experiments where the synthetic glucocorticoid dexamethasone (Dex) (Steraloids) was used, the cells were incubated with charcoal-stripped FBS (Hyclone) for the last 24 h before the stimuli.

### *E. coli* Strains

The CD2-a *E. coli* strain was previously isolated from a patient with CD, showing AIEC features as previously described (11). AIEC strains NRG857c and HM605, and control strain HB101 (38) were used (for strain characteristics see respective citations). Briefly, they are *E. coli* with heterogeneous virulence genes providing a higher survival rate within macrophages and invasion of epithelial cells. NRG857c and HM605 AIEC reference strains have been shown to be genetically heterogeneous (11). Strain HB101 was used to compare against an *E. coli* strain without a virulence phenotype. In addition, *S. aureus* subsp. aureus Rosenbach (ATCC^®^ 29213™) was used as a bacteria different to *E. coli* species. All strains were maintained at 37°C in RPMI medium overnight before each experiment was performed.

### RNA Isolation

Total RNA (over 1 µg) was isolated from THP-1 macrophages treated with 100 nM Dex and 100 µg/mL amikacin for 6 h, infected with CD2-a *E. coli* strain with a multiplicity of infection (MOI) of 1 for 2 h, later followed by 6 h amikacin incubation, or followed by 6 h amikacin and Dex incubation. The RNeasy^®^ Mini Kit (Qiagen) was used, according to manufacturer’s instructions. One million cells in 2 mL of complete RPMI 1640 medium were cultured in 12 well plates to obtain 100–1,000 ng/µL RNA.

### Microarray

Gene expression analysis was conducted using the Whole Human Genome 4 × 44 Multiplex oligonucleotide array (product no. 014850, Agilent Technologies). RNA samples at concentrations 0.2–0.5 µg/µL and OD 260/280 ratio 1.7–2.1 were used for this analysis. Cy3- labeled cRNA was fragmented and hybridized for 17 h in a rotation hybridization oven. Slides were scanned on an Agilent Scanner using Agilent Feature Extraction software version 12 (Agilent Technologies) to capture resulting data later analyzed with Omicsoft^®^ ArrayStudio^®^ version 7.0 software. Raw data were processed with Partek^®^ Genomic Suite^®^ software, version 6.6 Copyright©, 2016 Partek Inc., to generate a Principal Components Analysis and to find gene probes expressed with statistical significance. Venny free online software^1^ was used to generate Venn Diagrams for selected gene probes. Ingenuity^®^ Pathway Analysis (IPA^®^) version 6.5 tool (QIAGEN Inc.) (Ingenuity Systems)^2^ was used to identify functional pathways and their statistical significance. The canonical pathways figures were generated through the use of IPA^®^.

### Quantitative Real-Time PCR

Affinity script reverse transcriptase (Agilent) was used to generate cDNA from 1 RNA μg in 20 µL water, following the manufacturer’s instructions. Quantitative real-time PCR was performed from 2 µL cDNA in BIORAD CFX96 equipment using TaqMan assays for *CD14, CD48, MARCKS, GILZ*, and *PPIB* mRNA.

### Luminex^®^ Assay

THP-1 Cells (520,000/well in 24-well plates) were infected with *E. coli* strain CD2-a or left uninfected in 520 µL of RPMI 1640 complete medium without antibiotics for 2 h, followed by incubation with 100 µg/mL amikacin and treatments 100 nM dexamethasone or vehicle until 24 h post-infection. 25 µL of supernatant was used in a Luminex^®^ 200 assay (Merck). 20 cytokines and chemokines were selected from the panel kit HCYTMAG-60K and process according to manufacturer instructions.

### ELISA

Levels of TNF-α, IL-6, IL-12, IL-23, and IL-10 in supernatant from THP-1 macrophage cell cultures were determined by ELISA (R&D Systems). Cells (520,000/well in 24-well plates) were infected with *E. coli* strains in 1 mL of RPMI 1640 complete medium without antibiotics for 2 h, followed by 100 µg/mL amikacin and then by incubation with 100 nM dexamethasone, 1 µM RU486, co-treatment or vehicle until 24 h post-infection. Direct supernatant (100 µL) and diluted samples (20–50 times) were analyzed using ELISA plates. Cytokine concentrations were assessed according to the manufacturer’s instructions.

### Flow Cytometry

THP-1 macrophages (1 million cells/well in 12-well plates) were used in RPMI 1640 complete medium (2 mL, without antibiotics) were infected with *E. coli* strains CD2-a, NRG857c, HM605, and HB101 for 2 h, followed by incubation with 100 µg/mL amikacin and 100 nM dexamethasone, 1 µM RU486, co-treatment or vehicle until 24 h post-infection. After incubation, macrophages were surface-stained with fluorochrome-specific antibodies for human CD40, CD80, CD86, CD163 (all from BioLegend) and MHC class II molecules (eBioscience) or fixed and permeabilized for CD68 (BioLegend) intracellular staining. Prior to staining, cells were treated with FcR Blocking Reagent (Miltenyi) to avoid unspecific FcR staining. The viability of cell cultures was assessed using annexin-V and 7-AAD apoptosis detection kit (BD Pharmigen), following the manufacturer’s instructions. For each analysis, data from at least 5,000 cells were acquired.

### Lactate Dehydrogenase (LDH) Assay

Lactate dehydrogenase was detected in the supernatant of cell cultures using the CytoTox 96^®^ assay (Promega), following the manufacturer’s instructions.

### Giemsa Stain

Giemsa stain was used to assess the number of infected macrophages with different MOI of *E. coli*. Briefly, THP-1 macrophages were treated with *E. coli* strains for 1–2 h at 1–10 MOI in complete medium without antibiotics, followed by 100 µg/mL amikacin treatment for 1 h. Cells were washed with PBS and fixed with 70% methanol (Merck) for 7 min. Cells were stained with 5% Giemsa (Merck) in distilled water for 40 min. Slides were observed by optical microscopy, and infected cells were counted in a total of 200 cells.

### Western Blot Analysis

Protein from THP-1 monocytes and macrophages was extracted through sonication in Laemli buffer. Samples were boiled for 5 min and separated on a sodium dodecyl sulfate-10% polyacrylamide gel electrophoresis (Biorad). Proteins were transferred to a nitrocellulose membrane in a Biorad Trans-blot Turbo starter system. Odyssey blocking buffer (LICOR) was used to block for 1 h. Membranes were incubated overnight at 4°C with anti-actin and anti-glucocorticoid receptor antibodies with 1:10,000 and 1:2,000 dilutions (Cell Signaling), respectively. This was followed by secondary antibodies, anti-mouse and anti-rabbit, diluted 1:15,000 and incubated for 2 h for visualization by chemoluminescence in a LICOR Odyssey Imager. Semiquantification of immune-reactive bands was performed using Image J version 1.47 software (National Institute of Health).

### Amikacin Protection Assay

For the phagocytosis assay, THP-1, WT, and *Nod2*−/− macrophages were treated with 100 nM Dex, 1 µM RU486, as co-treatment or vehicle for 24 h. Then, macrophages were infected with *E. coli* strains CD2-a, NRG857c, HM605, and HB101 (MOI = 10) for 30 min, followed by incubation with 100 µg/mL amikacin (Sigma) for 1 h to eliminate extracellular bacteria. Macrophages were lysed for 5 min in 1% Triton X-100 (Biorad) in PBS. Lysates were serially diluted and plated on lysogeny broth agar plates (Merck). After overnight incubation at 37°C, colonies were quantified. For bactericidal activity assay, the procedure was performed as stated above except that macrophages were infected for 2 h, followed by incubation with amikacin between 8 and 72 h post-infection.

### Invasion Assay

The invasion assays were performed on Caco-2 cells cultured in 24-well plates in DMEM without antibiotics and kept in 5% CO_2_ at 37°C. The cell monolayer was infected with strains HB101 and CD2-a *E. coli*, using a MOI of 10 for 3 h at 37°C. After this incubation period, cells were washed with PBS and incubated with fresh medium supplemented with Amikacin (100 µg/µL) for 3 h in 5% CO_2_ at 37°C. After this time, the cells were lysed with Triton-X-100 at 0.1% in PBS. Serial dilutions of these lysates were seeded on LC agar plates and incubated at room temperature overnight. The colonies were counted the next day, and the invasion percentage was determined as CFU/mL per hour post-antibiotic treatment in relation to the initial inoculum.

### Virulence Gene Analysis

Whole genome sequencing of CD2-a *E. coli* strain was performed with the HiSeq 2000 platform (Illumina) using the paired-end method. *De novo* assembly was performed with SPADES version 3.8.1 (39), using a pre-assembly approach with Velvet version 1.2.09 (39). This Whole Genome Shotgun project has been deposited at DDBJ/ENA/GenBank under the accession PDWX00000000. The version used in this paper is version PDWX01000000. To obtain genome annotation, PATRIC software version 3.4.12 (40) was used. Virulence genes were listed and compared with the ones from public reference *E. coli* strain genomes.

### Statistical Analysis

ANOVA was performed to determine significant differences between gene probes obtained from whole human mRNA microarray assay. A *p*-value of ≤0.05 and a cutoff of 1.5-fold change was used to select gene probes suitable for studying. ANOVA and Student’s *t*-tests were used to compare treatments between control or infected macrophages for the amikacin protection assay, ELISA, and flow cytometry results. Bonferroni and Dunnet post-tests were used for ANOVA analysis. Spearman rank coefficient was calculated for correlation of mRNA and soluble protein. All analyses were performed in GraphPad Prism 7 software (GraphPad Software Inc.).

## Results

### CD2-a *E. coli* and Dexamethasone Modulate Inflammatory and Bactericidal Gene Expression of THP-1 Macrophages

**Figure 1:**
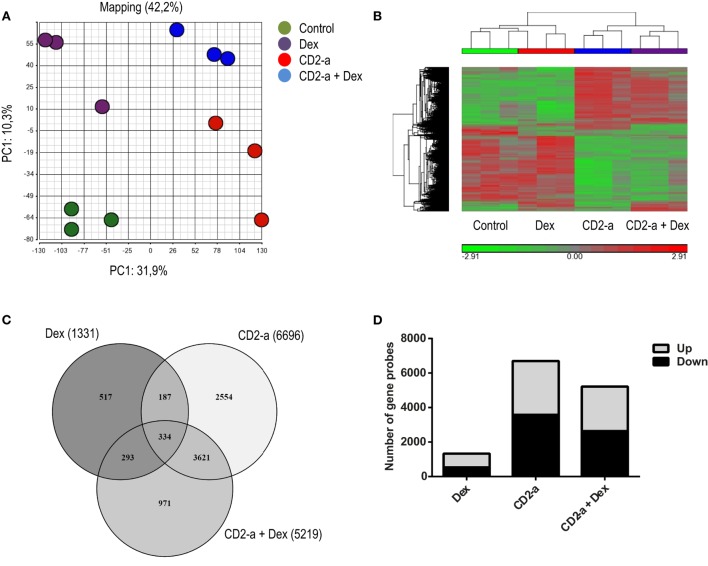
Dex and CD2-a *Escherichia coli* strains modulate gene expression in THP-1 macrophages. **(A)** Principal component analysis (PCA) from mRNA microarray performed on THP-1 macrophages. PCA shows the two main components, with CD2-a exerting more influence on variance as compared to Dex. **(B)** Cluster analysis of normalized samples. ANOVA (*p* < 0.05) and a cutoff of 1.5-fold change were used to select probes with statistical significance. Hierarchical clustering was performed based on 8,477 selected gene probes. **(C)** Venn diagram of the overlap in significant gene probes regulated by infection and/or Dex treatment. **(D)** Number of gene probes either induced (up) or repressed (down) by each condition.

In order to identify genes and pathways modulated by glucocorticoids on uninfected and infected macrophages, we first compared the transcriptional profiles of THP-1 macrophages (control), Dex-treated (Dex), CD2-a *E. coli* infected (CD2-a), and CD2-a *E. coli* infected + Dex-treated (CD2-a + Dex) THP-1 macrophages (Figure S2 in Supplementary Material). We chose 100 nM Dex as it had the highest induction of glucocorticoid response element (*GILZ*) mRNA levels (Figure S3 in Supplementary Material). Principal component analysis was performed to give an overview of the reproducibility and impact of each treatment on variance. The main and second axis of variance revealed that the CD2-a *E. coli* strain and Dex impacted gene expression, with the latter to a lesser extent (Figure 1A). To visualize how gene expression is altered by each treatment regime, hierarchical clustering was performed (Figure 1B). A total of 8,477 gene probes were regulated by all interventions, with 1,331 gene probes modulated by Dex, while 6,696 were modulated by CD2-a infection. Interestingly, infection for 2 h with CD2-a followed by 6 h of Dex regulated 5,219 gene probes, 971 of which were not affected by either Dex or CD2-a alone. Also, 627 gene probes were regulated by treatment or infection with either Dex or CD2-a + Dex, while 3,955 were regulated by either CD2-a or CD2-a + Dex (Figure 1C). There were slightly more genes upregulated (802) than downregulated (529) by Dex. With CD2-a and CD2-a + Dex, there was nearly an equal number of upregulated and downregulated gene probes (Figure 1D).

The above results have led us to identify the top five up- and down Dex-modulated genes and pathways that may have an impact on the immune response of macrophages against CD2-a *E. coli s*train (Table 1). Dex upregulated the *SAA1* gene (encoding a major acute phase protein) which was also upregulated by CD2-a + Dex but not by CD2-a alone. The gene downregulated by Dex with the higher negative fold change was the monocyte chemoattractant protein-1, *MCP-1/CCL2*.

**Table 1:** Genes upregulated and downregulated by Dex.

Dex vs. control	Fold change	CD2-a + Dex vs. CD2-a	Fold change
**Upregulated**			
*SAA1*	51.9	*TSC22D3*	118.8
*TSC22D3*	45.7	*TRIM63*	89.5
*ZBTB16*	42.8	*SAA1*	67.1
*SPRY1*	30.7	*NNMT*	44.1
*ADRA2C*	27.4	*FSTL3*	43.3
**Downregulated**			
*CCL2*	−21.7	*CSF2*	−20.5
*CXCL6*	−10.1	*IL36G*	−14.6
*VCAM1*	−9.5	*IL1A*	−13.3
*GPR88*	−8.7	*OSM*	−12.2
*RRAD*	−8.1	*LIF*	−9.4

*The five genes with the highest positive and negative fold change are in order of magnitude*.

**Figure 2:**
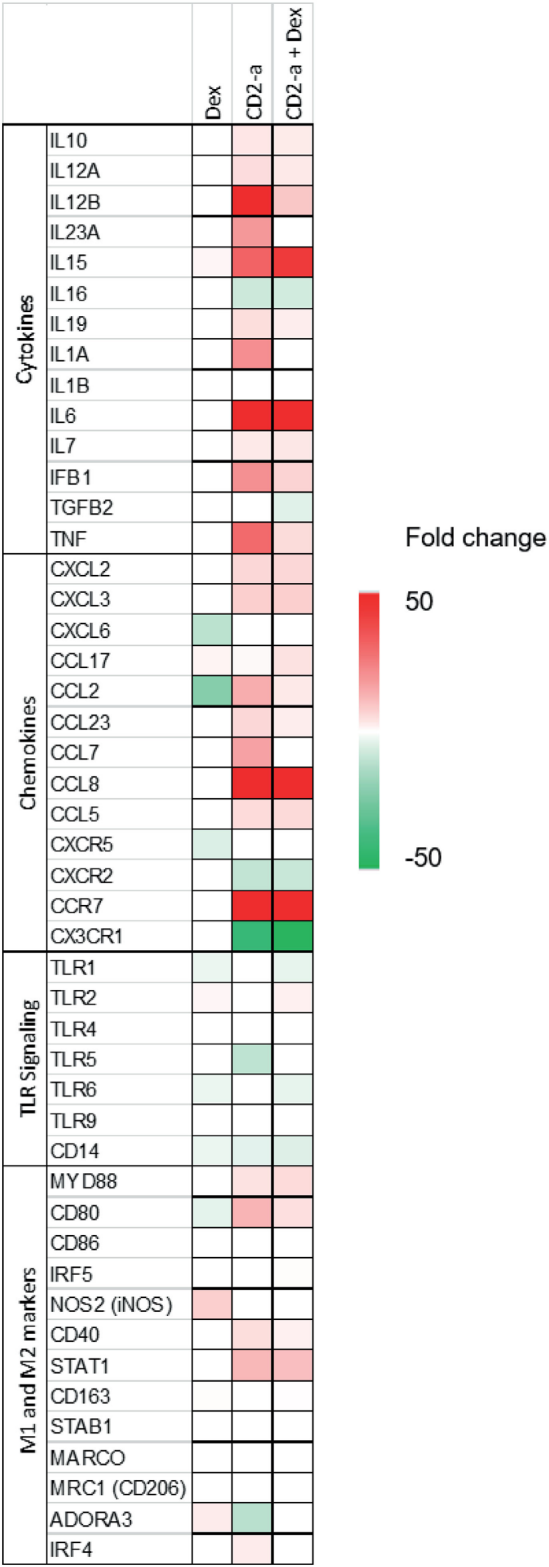
Dex and CD2-a modulated gene expression of multiple inflammatory genes. Genes that showed statistically significant differences for any of the three conditions and that affect immune response were selected and grouped into four categories: (1) cytokines, (2) chemokines, (3) TLR signaling, and (4) M1 and M2 markers. The heatmap indicates increased (red/upregulated) or decreased (green/downregulated) expression levels when subjected to each group, compared to control.

Inflammatory genes modulated by Dex treatment on uninfected or infected macrophages were selected in order to evaluate Dex’s ability to polarize macrophages to a M2-like phenotype (Figure 2). Cytokine genes were only downregulated by Dex in infected macrophages, and the two genes with the greatest fold change after CD2-a *E. coli* strain infection were *TNF* and *IL6*. Chemokines, such as *CXCL6* and *CXCR5*, were also downregulated by Dex. *TLR2* was upregulated by Dex, a phenomenon that has previously been described as one of the pro-inflammatory effects of glucocorticoid treatment (23, 41). Dex downregulated M1 markers in CD2-a *E. coli* strain infected macrophages (including *CD80* and *CD40*). However, an unexpected result was increased levels of iNOS gene *NOS2* in uninfected macrophages. Among M2 markers, few were upregulated by Dex, including *CD163* (42) and adenosine receptor A3 (*ADORA3*) (43) which was also upregulated in CD2-a + Dex-treated macrophages. The top five upstream regulators are listed in Table S1 in Supplementary Material.

**Figure 3:**
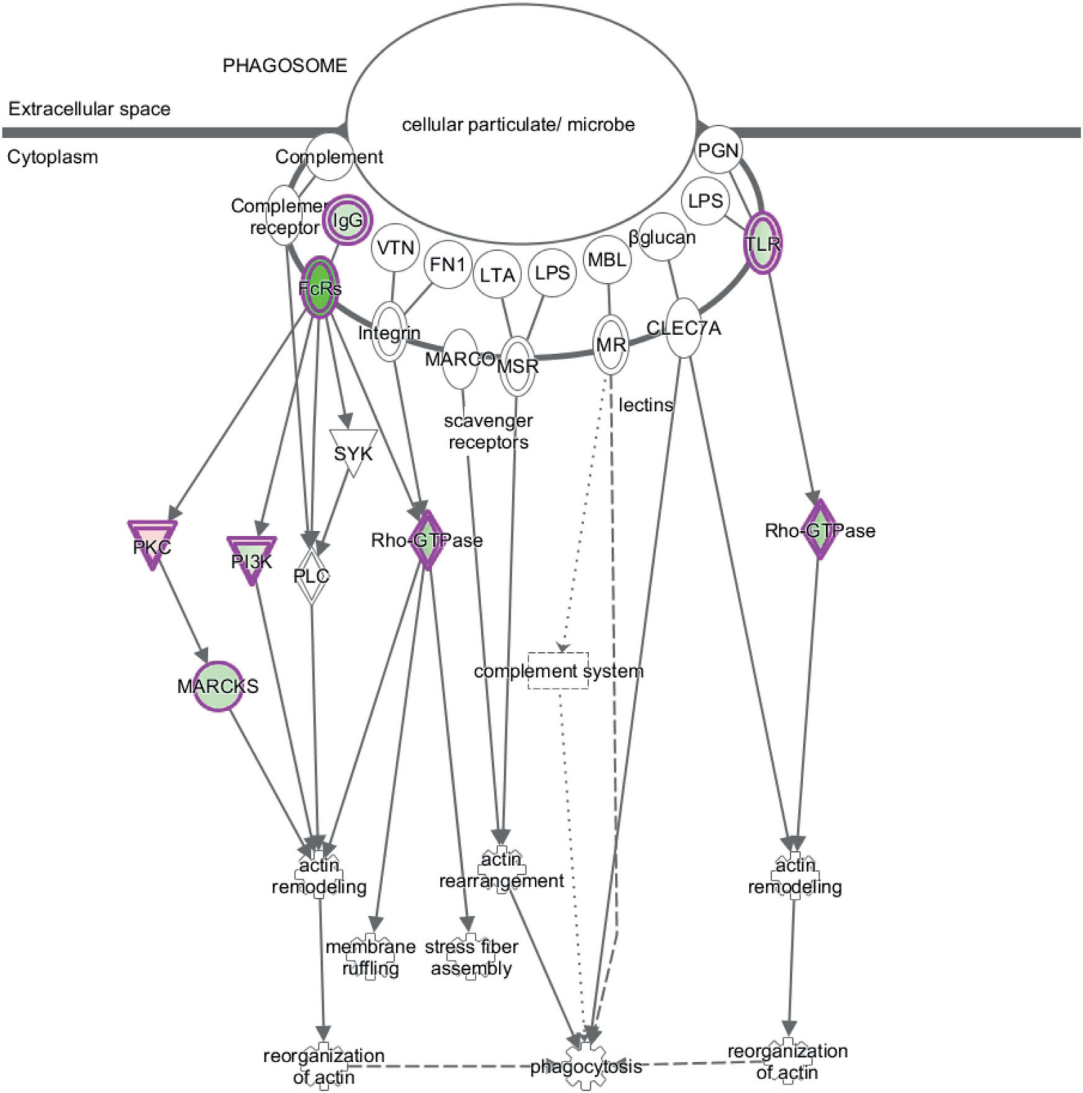
Dex represses expression of phagosome formation-associated genes. The figure shows the canonical pathway of phagosome formation according to the IPA database. Downregulated genes by Dex are highlighted in green and those upregulated in red.

Canonical pathway analysis has shown that Dex modulated the phagosome formation pathway (Table 2), while on CD2-a *E. coli* infected macrophages, Dex-modulated inflammatory pathways, such as NF-κB and TLR signaling. Figure 3 shows the phagosome formation canonical pathway according to the IPA database. Most genes were downregulated, as shown in green, which may imply an impairment.

**Table 2:** Top canonical pathways modulated by Dex.

	*p*-Value	Overlap
**Dex vs. control**		
Role of macrophages, fibroblasts, and endothelial cells in rheumatoid arthritis	1.08E−08	11.8% 35/296
G-protein coupled receptor signaling	1.47E−07	11.7% 30/256
Phagosome formation	3.15E−07	16.5% 18/109
Gq signaling	1.73E−06	13.6% 20/147
Glioma invasiveness signaling	2.11E−06	21.1% 12/57
**CD2-a + Dex vs. CD2-a**		
Role of macrophages, fibroblasts, and endothelial cells in rheumatoid arthritis	7.41E−05	10.8% 32/296
Toll-like receptor signaling	1.02E−04	17.6% 13/74
HMGB1	1.55E−04	14.2% 17/120
NF-kB signaling	2.60E−04	12.1% 21/173
FXR/RXR activation	3.10E−04	13.4% 17/127

Further microarray analysis validation was achieved by looking at secretion of cytokines and chemokines associated with immune response on the supernatant of THP-1 macrophages through a Luminex^®^ assay. Table S2 in Supplementary Material shows fold change of soluble proteins of and fold change of mRNA from Dex, CD2-a, and CD2-a + Dex treatments compared to control. Chemokines such as CCL2 and CXCL1 were downregulated by Dex as seen in the supernatant, similar to which was found in microarray data. Dex also downregulated expression of cytokines such as IL-6 and TNF-α on supernatant from THP-macrophages infected with CD2-a *E. coli* strain as also seen in mRNA levels in microarray analysis. Correlation analysis was performed between fold change of mRNA and soluble protein levels using Spearman’s test (Spearman correlation coefficient: 0.7582, *p* < 0.0001) according to what has been described in references (44).

### Dexamethasone Impairs THP-1 Macrophage Phagocytosis Against Bacteria

**Figure 4:**
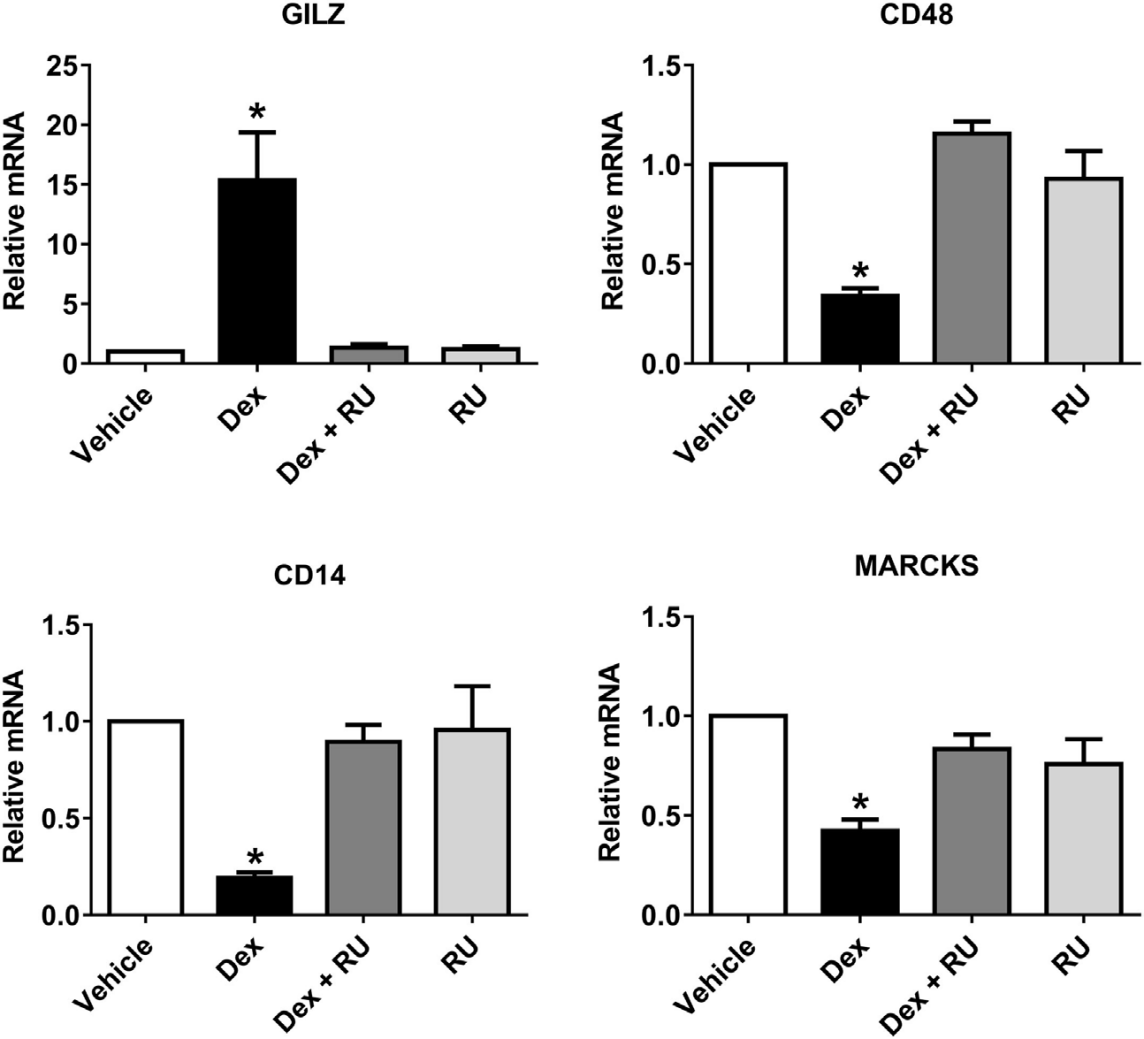
Quantitative PCR validation of Dex repressed phagocytosis-associated genes identified by microarray analysis. Three genes were selected by microarray from phagocytosis-associated genes that were also repressed by Dex: *CD48, CD14*, and *MARCKS*. Quantitative PCR was performed on macrophages treated with either 100 nM of Dex, 1 µM of the GC receptor inhibitory molecule RU486 or both, in order to evaluate whether the observed effects are due to GC receptor action. *GILZ*, a GC receptor response element, was used as positive control for GC receptor activation. One-way ANOVA and Bonferroni corrections were performed (*n* = 4; *p* < 0.05 compared to vehicle).

**Figure 5:**
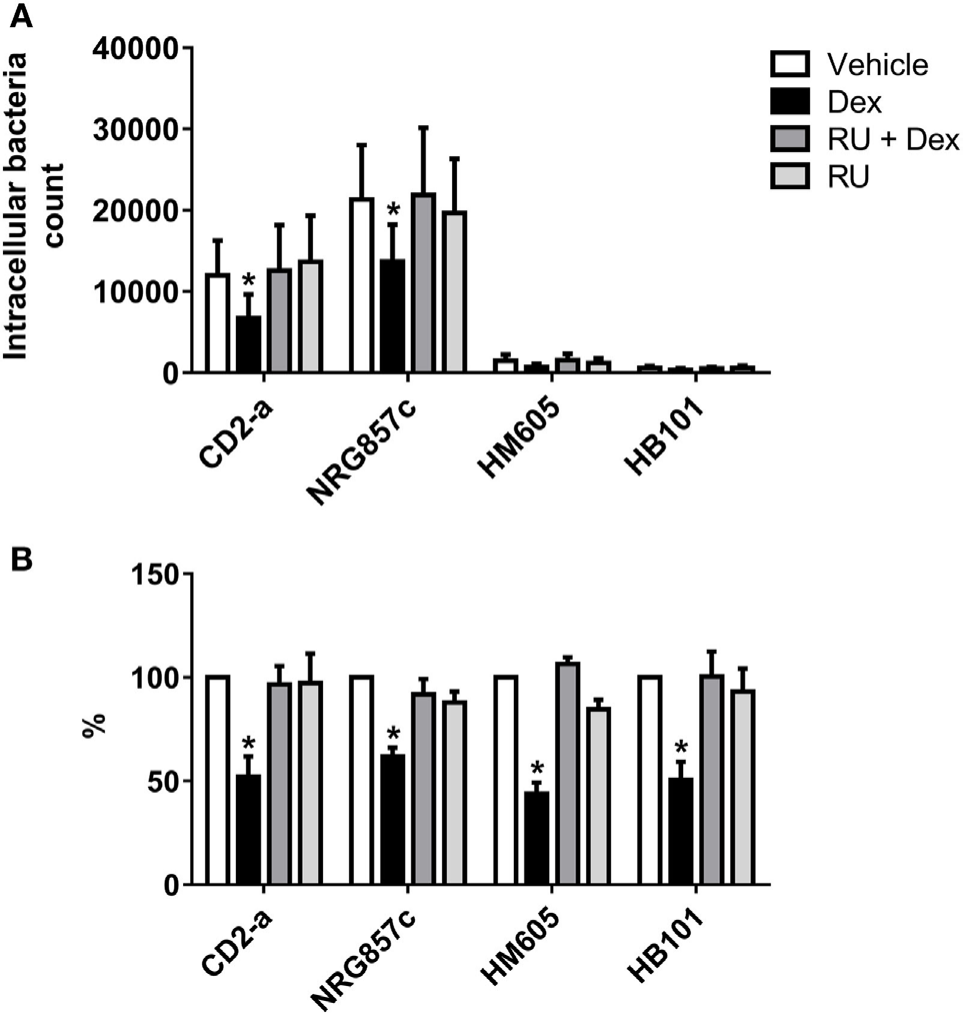
Dexamethasone decreases phagocytosis of macrophages against *Escherichia coli* strains. THP-1 macrophages treated with 100 nM Dex for 24 h were infected with *E. coli* strains (multiplicity of infection: 10). Amikacin protection assay was performed after 30 min of infection to evaluate phagocytic levels. **(A)** Absolute intracellular CFU count showing different quantities of phagocytosed strain inhibited by Dex. **(B)** Normalization to vehicle. Two-way ANOVA and Bonferroni corrections were performed (*n* = 6; *p* < 0.05 compared to vehicle).

**Figure 6:**
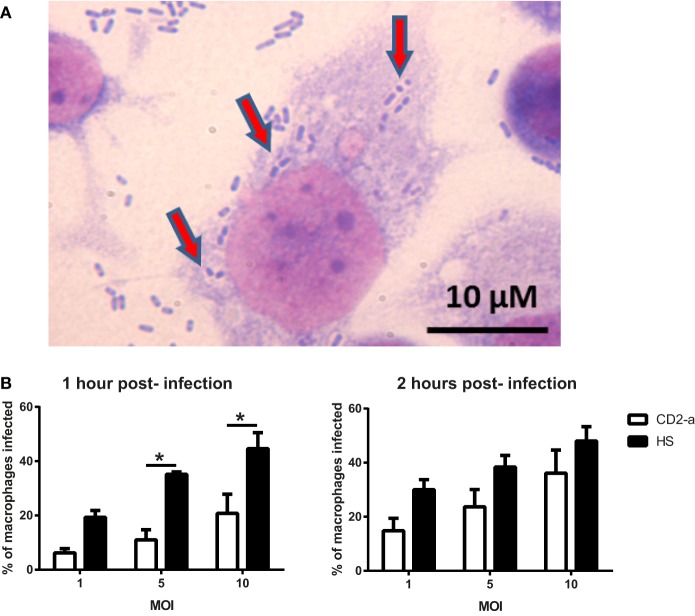
THP-1 macrophage infection depends on the multiplicity and duration of infection. Giemsa stain was used to determine bacterial counts inside macrophages. **(A)** Representative image of THP-1 macrophages infected with strain CD2-a. Red arrows show what appear to be *Escherichia coli* inside vacuoles in the cytoplasm. **(B)** Quantification of presumed infected macrophages shows that the level of infection (as a proportion) is dependent on the multiplicity and duration of infection. One-way ANOVA and Bonferroni corrections were performed (*n* = 3; *p* < 0.01).

In order to evaluate if phagosome formation was impaired *in vitro*, we selected three genes necessary for its formation that were downregulated by Dex in resting macrophages, namely *CD48* (17) (fold change: −5.83), *CD14* (18) (fold change: −2.00), and *MARCKS* (45) (fold change: −1.91). Their gene expression was validated by quantitative PCR, with results showing that all genes were downregulated by Dex with this effect being reversed by the glucocorticoid receptor inhibitor RU486, demonstrating glucocorticoid receptor signaling (Figure 4). Next, we evaluated the number of AIEC strains (CD2-a, NRG857c, and HM605) within Dex-treated macrophages for 24 h prior to infection by performing an amikacin protection assay, after phagocytosis has started (46) (Figure 5A). We previously evaluated the ability of THP-1 macrophages to acquire *E. coli* strains through a Giemsa stain (Figure 6) and found that using a MOI of 10 resulted in the highest number of infected macrophages. After Dex treatment, fewer CFUs were found intracellularly in macrophages, as compared to RU486-treated macrophages and when normalizing to the control condition, we found that Dex decreased CFUs by approximately 50%, independent of strain (Figure 5B), indicating that phagocytosis may be impaired. In order to evaluate if Dex could modulate macrophage death, LDH levels were measured in the supernatant which showed no difference compared to vehicle control (Figure S4 in Supplementary Material).

To assess whether the Dex-induced reduction in CFUs in *E. coli* infected macrophages was due to impairment of phagosome formation signaling or associated to change in bactericidal activity within the phagolysosome, an amikacin protection assay was performed until 72 h of infection. By normalizing CFU counts to the 3 h post-infection, macrophages were able to eliminate AIEC strains CD2-a, NRG857c, and HM605 at 72 h post-infection, with the HB101 strain easily eliminated 24 h post-infection. Dex had no effect on CFU count at any time compared to vehicle control, indicating that bactericidal activity was not affected (Figure S5 in Supplementary Material).

### Dexamethasone Impairs WT Murine Macrophage Phagocytosis Against Bacteria but Not in *Nod2*−/− Macrophages

**Figure 7:**
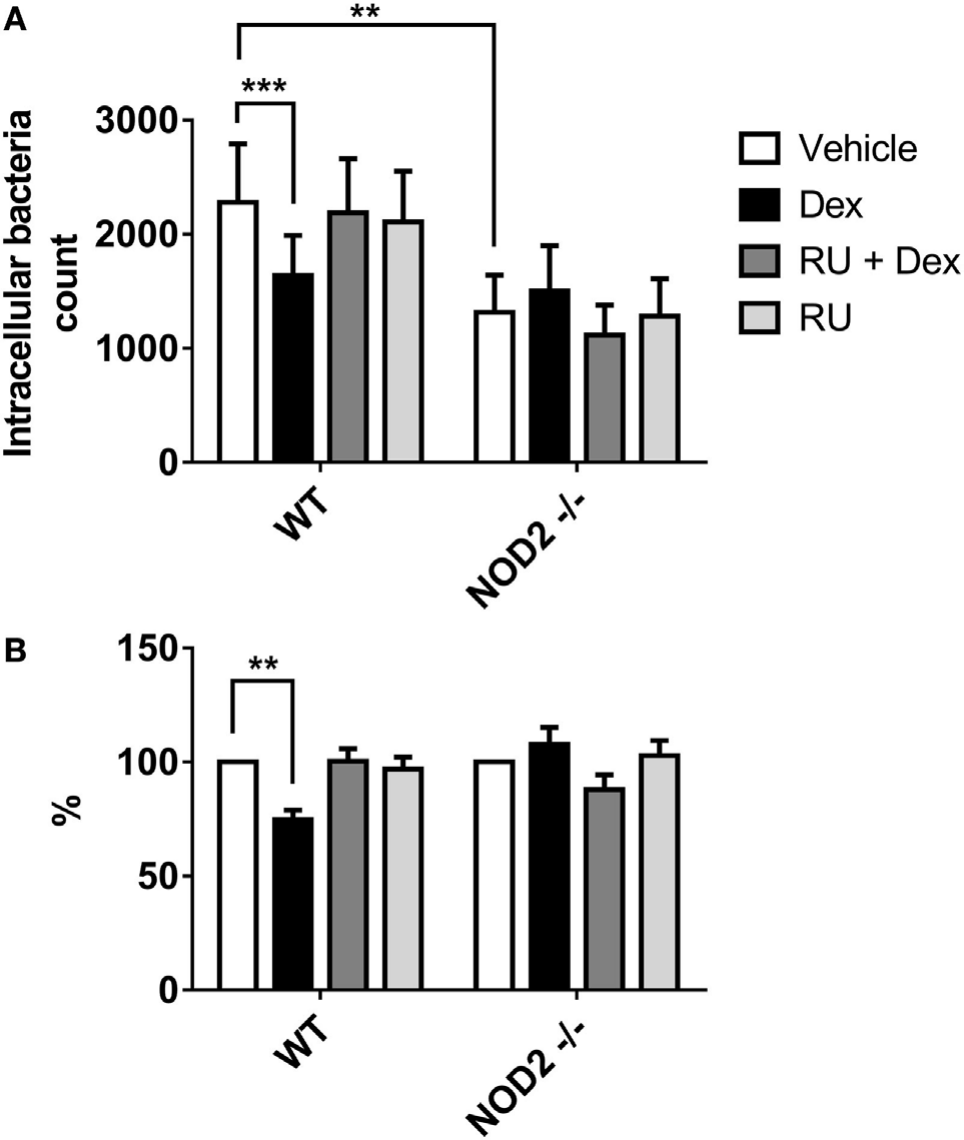
Dexamethasone decreases phagocytosis of murine macrophages against CD2-a *Escherichia coli* strain, but not on *Nod2*−/−. Wild type (WT) and *Nod2*−/− macrophages treated with 100 nM Dex for 24 h were infected with *E. coli* strains (multiplicity of infection: 10). Amikacin protection assay was performed 30 min after infection to evaluate phagocytic levels. **(A)** Absolute intracellular CFU count. **(B)** Normalization to vehicle. Two-way ANOVA and Dunnet corrections were performed (*n* = 5; ***p < 0.001 compared to vehicle). Student’s *t*-test was performed to compare absolute numbers between WT and *Nod2*−/− (*n* = 5; **p < 0.01).

In order to evaluate the significance of AIEC in IBD pathophysiology, we investigated glucocorticoids effect on the phagocytosis of AIEC in macrophages derived from mice lacking *Nod2*. Murine WT and *Nod2*−/− macrophages were used in amikacin protection assay and infected with CD2-a *E. coli* strain to determine phagocytosis modulation by Dex. Figure 7 shows that Dex decreased phagocytosis on WT macrophages. Surprisingly, phagocytosis in *Nod2*−/− macrophages was not modulated by Dex in terms of absolute or normalized number of CFUs compared to vehicle. This unexpected result might be due to *Nod2*−/− macrophages showed lower phagocytosis activity compared to WT macrophages, suggesting that *Nod2* may be necessary for correct phagocytosis.

### Dexamethasone Does Not Alter Epithelial Cell Susceptibility to AIEC Invasion

To evaluate the glucocorticoid effect on AIEC infection on another component of the mucosa, such as epithelial cells, Caco-2 cell monolayer model of the intestinal barrier was used. Dex modulation of epithelial cells susceptibility to AIEC infection was evaluated by an invasion assay on Caco-2 cell line with CD2-a and HB101 *E. coli* strains. After 3 h, Dex failed to modify the intracellular number of bacteria (Figure S6 in Supplementary Material) showing that epithelial cell susceptibility to AIEC invasion is not influenced directly by glucocorticoids.

### Dexamethasone Inhibits Inflammatory Response of THP-1 Macrophages Against AIEC

**Figure 8:**
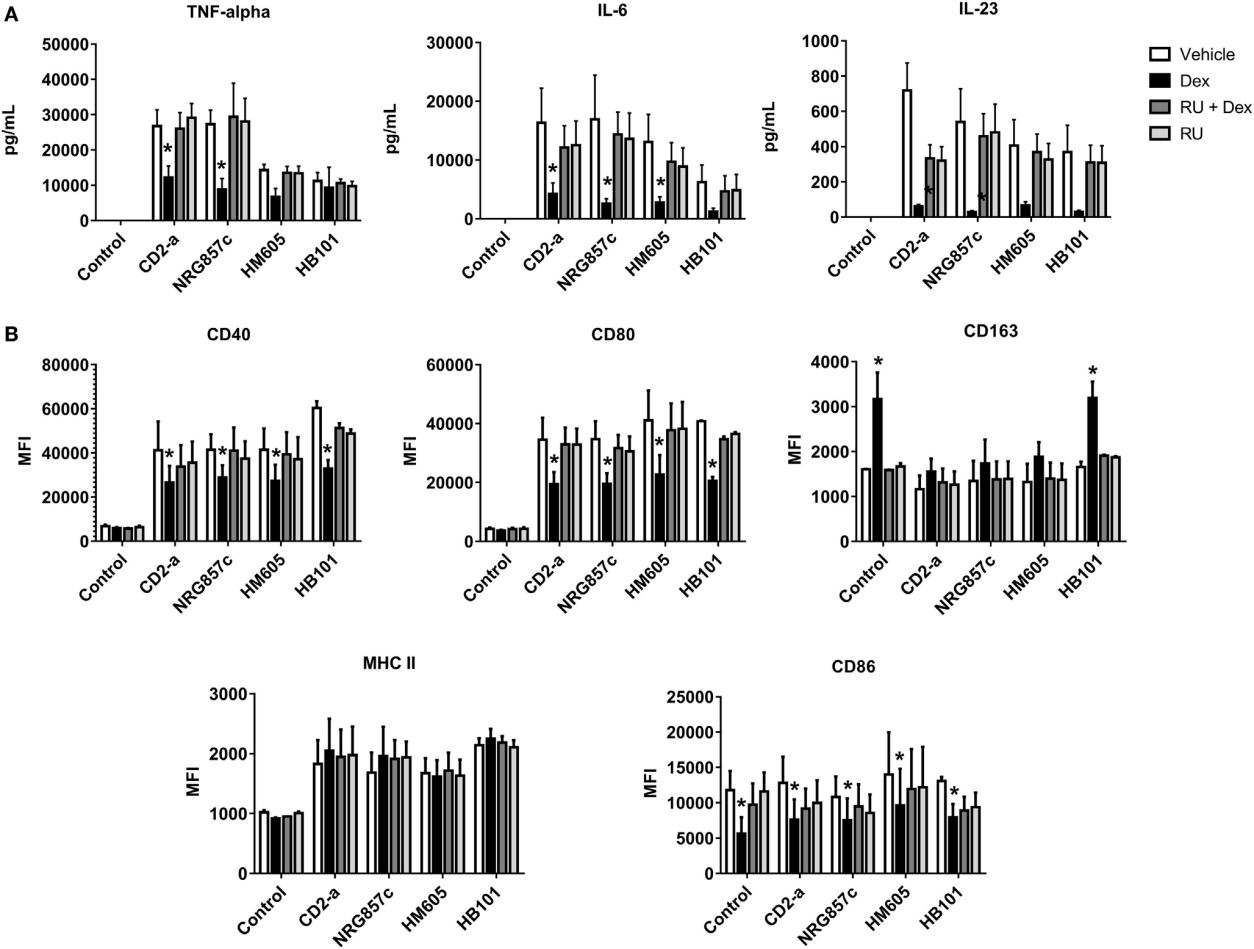
Dexamethasone decreases inflammatory markers in *Escherichia coli*-treated macrophages. THP-1 macrophages were infected with *E. coli* strains for 2 h, followed by Dex and amikacin treatment as described in the Section “Materials and Methods.” ELISA **(A)** and flow cytometry assays **(B)**. Two-way ANOVA and Bonferroni corrections were performed (*n* = 5; *p* < 0.05 compared to vehicle).

We showed that IL-23, TNF-α, and IL-6 were highly induced by all *E. coli* strains, with this phenomenon being reversed by Dex treatment from 2 to 24 h post-infection (Figure 8A) (IL-12 was not detectable in any of the conditions; data not shown), demonstrating that Dex is capable of reversing the M1 polarization of macrophages after infection. In addition, the inflammatory markers CD40, CD80, and MHC class II molecules were induced by all *E. coli* strains with Dex treatment decreasing CD40 and CD80 expression with no effect on MHC class II molecules (Figure 8B; gating strategy on Figure S7 in Supplementary Material). Furthermore, *E. coli* did not induce CD86, but its expression was decreased after Dex stimuli, even on macrophages without infection. Interestingly, Dex increased CD163 levels in HB101-infected and not infected macrophages, while no effect on AIEC infected macrophages was recorded.

In addition, the whole genome of the CD2-a *E. coli* strain was sequenced, allowing us to evaluate differences in virulence genes on each AIEC strain and propose relevant candidates in the interaction between glucocorticoids and macrophages. While comparing CD2-a *E. coli* strain against the four reference AIEC strains: NRG857c, HM605, LF82, and UM146 together with commensal strains (HS and K-12 substrain MG1665), no distinctive virulence gene profile associated with AIEC bacteria were discovered as previously described (47, 48) (Table S3 in Supplementary Material).

## Discussion

At present, it is unknown if glucocorticoid treatment alters the outcome of AIEC infection on macrophages, so understanding this process could help to improve CD treatment. Here, we show that the glucocorticoid dexamethasone can significantly modify how macrophages, derived from THP-1 cells, interact with adherent-invasive and control *E. coli* strains in terms of inflammatory response and phagocytic activity. Microarray data show that THP-1 macrophages respond to Dex by activating pathways associated with glucocorticoid receptor activation and that Dex can differentially regulate gene expression profiles depending on the initial inflammatory state of the macrophage. On uninfected and CD2-a *E. coli* strain infected macrophages, the top five upregulated genes with the highest fold change included the anti-inflammatory transcription factor *TSC22D3* (*GILZ*). As expected, this protein, a well-known element of response to glucocorticoid treatment, can attenuate inflammation and promote tolerance against LPS (49). Also, the acute phase protein *SAA1* was upregulated by Dex in both resting and infected macrophages, which is consistent with previous studies (50, 51). Aggregates of *SAA1* can be seen in many inflammatory diseases, including CD, resulting in a condition called amyloidosis (52). Previous studies have pointed out the value of considering glucocorticoid treatment in cases of inflammatory disease-associated amyloidosis (53), and our results add weight to this debate by showing that glucocorticoids may increase *SAA1* levels in macrophages contributing to amyloidosis in CD. More studies on this relationship are needed.

On uninfected macrophages, *CCL2* and *CXCL6* were among the top five downregulated genes, shown by microarray and with CCL2 confirmed by Luminex^®^ analysis. CCL2 is a strong chemoattractant for monocytes and macrophages and implicated in granuloma development, a known sequela of CD inflammation (54). Whether glucocorticoids help in the prevention of granuloma development by decreasing CCL2 levels remains unknown. In addition, CXCL6 is also a strong chemoattractant for granulocytes (55). These findings suggest that recruitment of macrophages and neutrophils to the tissue may be decreased under glucocorticoid treatment. On CD2-a *E. coli* strain infected macrophages, Dex-induced downregulation of *CSF2* and the cytokine genes *IL36G* and *IL1A*, which suggest that glucocorticoid may impair inflammatory response against bacteria. On canonical pathway analysis, it is seen that phagosome formation is regulated by Dex, mainly by downregulation. Through quantitative PCR it was confirmed that Dex downregulates mRNA levels of at least three phagocytosis-associated genes (*CD14, CD48*, and *MARCKS*), whose products are involved in different aspects of the phagosome formation pathway: CD14 acts as a co-receptor for TLR2- and TLR4-associated phagocytosis (18), CD48 as a bacterial fimH receptor (17), and MARCKS as an actin-cell membrane anchor protein (45). Based on our results, we propose that glucocorticoid impairs phagocytosis by acting at different levels within the phagosome pathway, rather than by impairing a single step or protein.

*In vitro* assay confirm that Dex impairs phagocytosis of commensal *E. coli* and AIEC strains. Since AIEC strains have invasion abilities, HB101 was used to compare phagocytosis activity against an *E. coli* strain that is engulfed by macrophages through phagocytosis and does not have invasion activity. By using *S. aureus*, we also demonstrated that glucocorticoid effect on phagocytic activity was not specific to one type of bacteria, as *S. aureus* phagocytosis was impaired at similar levels as for *E. coli* (Figure S8 in Supplementary Material).

When evaluating glucocorticoid on *Nod2*−/− murine macrophages as a CD model, we found that Dex decreases phagocytosis in WT but not in *Nod2* deficient cells. However, intrinsic phagocytosis was found to be lower in *Nod2*−/− than WT macrophages. Although NOD2 is necessary for phagocytosis in murine neutrophils, it has not been studied on macrophages (36). One possible explanation of the phagocytosis impairment by glucocorticoids is that it could be a NOD2-dependent mechanism, which occurs in THP-1 cells and murine WT but not on *Nod2*−/− macrophages. This means *Nod2*−/− macrophages are not a suitable model for studying glucocorticoid modulation of phagocytosis. This suggests that patients carrying a defective NOD2 function would show phagocytosis impairment, and thus, glucocorticoid treatment would be obsolete. However, in patients with functional NOD2, glucocorticoid treatment may be harming phagocytosis, thus disturbing bacterial clearance.

Glucocorticoid impairment of phagocytosis may not only be an issue in CD but in all inflammatory pathologies where they are used as treatment, thereby increasing infection risk. Several authors have evaluated the effects of glucocorticoids on phagocytosis, and their results have been contradictory to each other (29, 32–34). One possible explanation is that there is no standardization for phagocytosis assays, as different elements of phagocytosis can be used and macrophages can be obtained from tissues or *in vitro* differentiated cell cultures.

Glucocorticoids also polarize infected macrophages into an anti-inflammatory phenotype, by decreasing CD40, CD80, and CD86, thus impairing T cell activation (20). As evidenced by the reduction in supernatant levels of IL-6, TNF-α, and IL-23 from CD2-a infected macrophages compared to bacterial infection without Dex, glucocorticoids appear to affect inflammatory cytokine secretion. TNF-α can induce the expression of CEACAM6 in epithelial cells, which is a receptor used by AIEC to adhere to the epithelium (56). Glucocorticoids could impair adhesion of AIEC to epithelial cells indirectly by decreasing TNF-α levels, and thus CEACAM6 expression.

**Figure 9:**
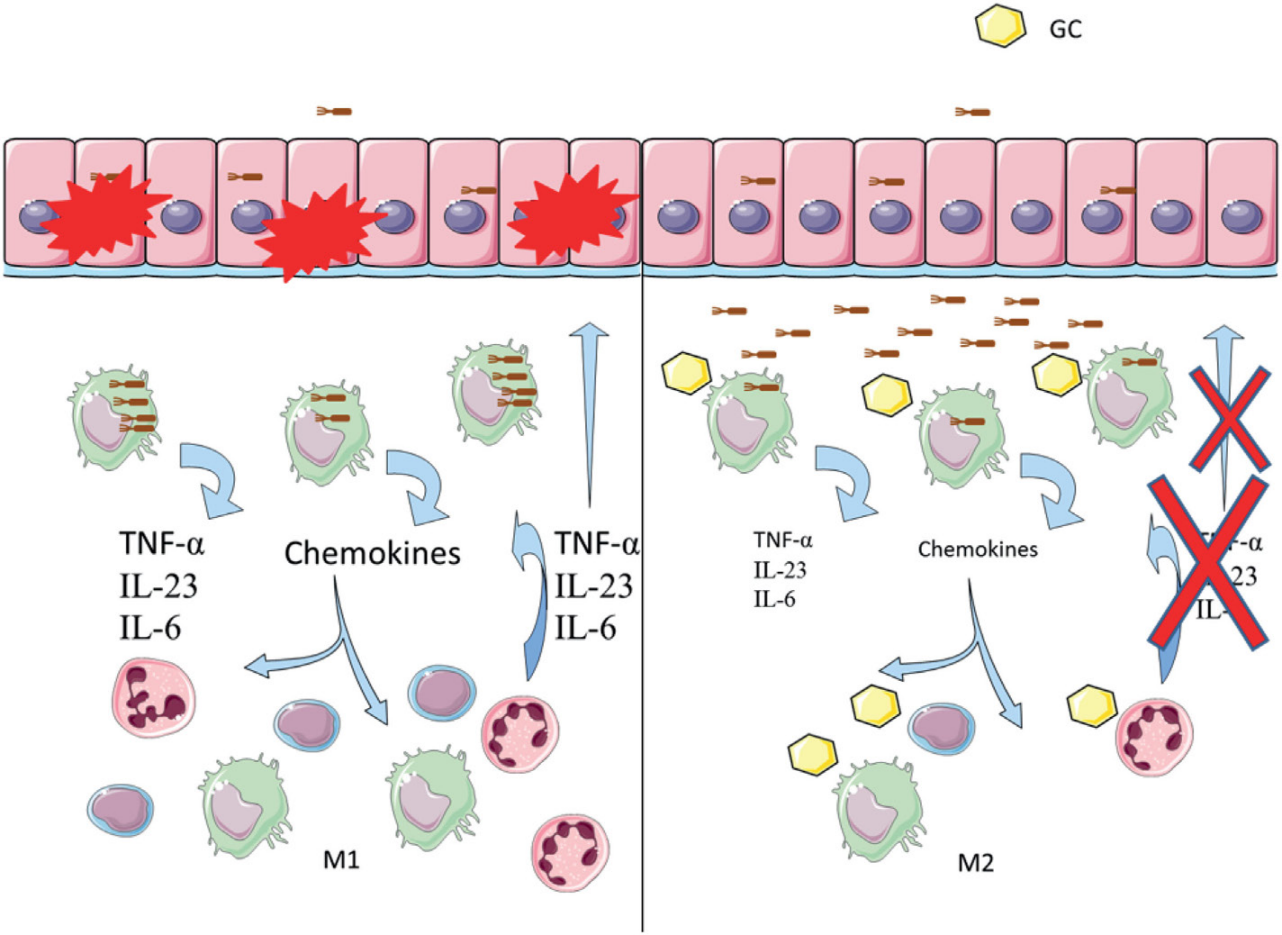
Glucocorticoids induce inhibition of phagocytosis in Crohn’s disease (CD). Left: the proposed model of disease indicates that bacteria such as adherent-invasive *Escherichia coli* (AIEC) may trigger or maintain inflammation in the gut mucosa. AIEC can infect and cross the epithelial barrier, being afterward phagocytosed by residing macrophages. Increased survival of AIEC inside macrophages induces secretion of chemokines and cytokines. Immune cells are recruited, increasing the inflammatory response and epithelium damage. Right: proposed model of glucocorticoid inducing chronicity in CD. Glucocorticoid treatment may decrease phagocytosis of AIEC by macrophages, impairing bacterial clearance. Decreased inflammatory response affects cell recruitment, which may then die through glucocorticoid-induced apoptosis. A low-grade inflammatory environment protects from epithelium damage but increases AIEC’s chances of survival, which likely perpetuates the disease.

Given the anti-inflammatory environment generated by glucocorticoids, impaired phagocytosis could mean that bacteria will be abundant in the mucosa without causing inflammation, and the effect of glucocorticoid treatment could then support AIEC colonization and CD. In Figure 9, we propose a model for how glucocorticoid might be promoting AIEC bacteria to survive asymptomatically in the intestinal mucosa.

We believe that at present the THP-1 macrophage appears to be a good cell model for studying macrophage interactions, together with *Nod2*−/− cells, and offering a better first step in understanding the impact of glucocorticoids on AIEC infected macrophages and the resulting inflammatory response. In addition, it is important to determine if glucocorticoid impairs phagocytosis of AIEC bacteria in the gut using *in vivo* models. We have found previously that patients with IBD treated with glucocorticoids had more CFUs of *E. coli* in ileal mucosal tissue compared to controls (57), which possibly reflects the inability of glucocorticoid-stimulated macrophages to clear the bacterial infection in the intestinal mucosa.

## Conclusion

In conclusion, we determined that glucocorticoids decrease AIEC phagocytosis and diminish inflammatory marker levels in THP-1 macrophages. This condition may cause impairment of AIEC clearance from the gut mucosa in CD patients carrying a defective NOD2 gene, and therefore the chances of a chronic disease.

Moreover, many questions have arisen in regard to phagocytosis and inflammation, which may be worth further study in the future, and are listed as follows: (a) AIEC survival, and CD complications; (b) components of phagocytosis that are modulated by glucocorticoids, such as MARCKS and CD14; (c) increased SAA1 levels associated with glucocorticoid treatment; and (d) the possibility that glucocorticoids can protect against granuloma formation by decreasing CCL2 levels.

Subsequent work is needed to clarify if the observed effects of glucocorticoids also occur in different macrophage phenotypes derived from the gut mucosa of CD patients. These studies will help physicians improve patient management and quality of life in the future.

## Author Contributions

MO-M performed most of the experiments and bioinformatics analysis, contributed to study design and drafting of the manuscript. MF contributed to study design, isolation of AIEC strain CD2-a, and drafting the manuscript. KD contributed to study design and manuscript discussion. DP performed some of the *in vitro* experiments and reviewed the manuscript. DD-J contributed to study design. AT-R performed some of the experiments and reviewed the manuscript. XX performed bioinformatics analysis. NC performed bioinformatics analysis, invasion assays, and reviewed manuscript. RN contributed to study design and supervised the work. M-JG contributed to some experiments and reviewed the manuscript. RQ contributed to study design and sample acquisition. CF contributed to study design and sample acquisition. JC contributed to study design, microarray analysis, supervised the work, and reviewed the manuscript. RV contributed to study design, supervised work, contributed with *E. coli* reference strains and funding. MH contributed to study design, supervised work, drafting the manuscript, and obtained funding.

## Conflict of Interest Statement

The authors of this work declare that the research was conducted in the absence of any commercial or financial relationships that could be construed as a potential conflict of interest.

## Footnotes

^1^http://bioinfogp.cnb.csic.es/tools/venny/ (Accessed: September 25, 2015).

^2^https://www.qiagenbioinformatics.com/products/ingenuity-pathway-analysis (Accessed: September 24, 2015).
