# Supplementary material for: Glucocorticoids Impair Phagocytosis and Inflammatory Response Against Crohn’s Disease-Associated Adherent-Invasive Escherichia coli
Source: Front Immunol. 2018 May 16;9:1026. doi: 10.3389/fimmu.2018.01026 (PMC5964128; doi:10.3389/fimmu.2018.01026)
Supplement: Supplementary file 1 [file table_1.PDF]

Supplementary Table 1. Upstream regulators modulated by Dex		
**Dex vs. Control**	***P*-value**	**Predicted activation**
Lipopolysaccharide	1.82E-22	
TGFB1	3.22E-20	
TNF	5.99E-19	
Triamcinolone acetonide	9.12E-18	Activated
Dexamethasone	6.71E-17	Activated
**CD2-a Dex vs. CD2-a**	***P*-value**	**Predicted activation**
TNF	1.18E-19	Inhibited
Triamcinolone acetonide	2.42E-17	Activated
U0126	3.54E-17	
NR3C1	1.20E-15	Activated
Salmonella enterica serotype abortus equi lipopolysaccharide	9.92E-15	Inhibited
