# Supplementary material for: Glucocorticoids Impair Phagocytosis and Inflammatory Response Against Crohn’s Disease-Associated Adherent-Invasive Escherichia coli
Source: Front Immunol. 2018 May 16;9:1026. doi: 10.3389/fimmu.2018.01026 (PMC5964128; doi:10.3389/fimmu.2018.01026)
Supplement: Supplementary file 2 [file table_2.PDF]

Suplemmentary table 2. Fold change of mRNA and soluble protein from THP-1 macrophages*							
		Dex		CD2-a		CD2-a + Dex	
Protein	Gene	mRNA	Soluble Protein	mRNA	Soluble Protein	mRNA	Soluble Protein
Eotaxin	*CCL11*	1.0	1.0	1.3	<-1011.4	1.1	<-1756.5
G-CSF	*CSF3*	1.5	-1.0	102.8	5.6	164.5	9.4
GM-CSF	*CSF2*	-3.7	1.0	53.1	23.6	2.6	3.6
IFN-α2	*IFNA2*	-1.0	1.1	-1.0	7.4	-1.0	6.0
GRO	*CXCL1*	-5.4	-1.0	2.6	706.8	1.7	458.5
IL-10	*IL10*	-1.1	-1.5	4.9	186.9	4.1	19.8
MCP-3	*CCL7*	-4.7	1.1	17.5	18.9	4.3	4.2
IL-12p40	*IL12B*	-2.0	-1.2	76.5	6.9	10.2	2.0
MDC	*CCL22*	-1.5	1.5	6.9	46.9	5.4	17.8
IL-12p70	*IL12A*	1.5	-1.2	6.4	2.8	4.5	1.6
IL-1RA	*IL1RN*	-3.0	-2.0	8.7	35.5	3.1	9.4
IL-1α	*IL1A*	-3.6	-1.3	22.1	26.4	1.7	14.6
IL-1β	*IL1B*	-4.5	-1.1	5.9	278.4	4.0	249.1
IL-6	*IL6*	-3.5	-1.1	699.0	728.8	104.2	111.7
IL-8	*CXCL8*	-4.7	-3.5	4.9	73.1	3.0	164.9
IP10	*CXCL10*	-1.7	-1.6	515.7	317.2	682.6	334.1
MCP-1	*CCL2*	-21.7	-2.7	15.3	>155.6	4.4	>155.6
MIP-1α	*CCL3*	-3.2	-1.7	3.2	>1693.4	3.5	>1693.4
MIP-1β	*CCL4*	-2.7	-1.4	4.3	>129.8	4.5	>129.8
TNF-α	*TNF*	-4.1	-1.4	30.9	584.2	6.5	284.1

r (Spearman): 0.7582

$p < 0.0001$

* Fold change from treated macrophages compared to Control. The analytes *CCL11*, *CCL2*, *CCL3*, and *CCL4* were excluded for analysis because values were out of the detection range
