# Supplementary material for: Glucocorticoids Impair Phagocytosis and Inflammatory Response Against Crohn’s Disease-Associated Adherent-Invasive Escherichia coli
Source: Front Immunol. 2018 May 16;9:1026. doi: 10.3389/fimmu.2018.01026 (PMC5964128; doi:10.3389/fimmu.2018.01026)
Supplement: Supplementary file 3 [file table_3.PDF]

Supplementary Table 2. Virulence genes of AIEC strains									
Identifier	Product/Function	Source Organism	CD2-a	NRG857c	HM605	LF82	UM146	HS	K12-substr. MG1665
**Antiphagocytosis/Invasion**									
rfaG	UDP-glucose:(heptosyl) LPS alpha1,3-glucosyltransferase WaaG	Salmonella enterica subsp. enterica serovar Enteritidis str. P125109	+	+	+	+	+	+	+
dsbA	Periplasmic thiol:disulfide interchange protein DsbA	Shigella flexneri 5 str. 8401	+	-	-	-	-	+	+
KpsD	Capsular polysaccharide export system periplasmic protein KpsD	Escherichia coli	-	+	+	+	+	-	-
KpsT	Capsular polysaccharide ABC transporter, ATP-binding protein KpsT	Escherichia coli	-	-	+	-	+	-	-
KpsM	Capsular polysaccharide ABC transporter, permease protein KpsM	Escherichia coli	-	+	+	+	+	-	-
ORF B protein	Transposase InsO for insertion sequence element IS911	Escherichia coli 536	+	-	+	-	-	-	+
L7045	Transposase InsN for insertion sequence element IS911	Escherichia coli 536	+	-	+	-	-	-	+
Z1203	Co-activator of prophage gene expression IbrA	Escherichia coli 536	-	-	+	-	+	-	-
Z1204	Co-activator of prophage gene expression IbrB	Escherichia coli 536	-	-	+	-	+	-	-
Z1215	UPF0380 proteins YafZ and homologs	Escherichia coli 536	-	-	+	-	-	-	-
Z1216	Antirestriction protein klcA	Escherichia coli 536	-	-	+	-	-	-	-

Z1217	UPF0758 protein YeeS	Escherichia coli 536	+	-	+	-	+	-	+
**Iron uptake**									
iroN	Outer Membrane Siderophore Receptor IroN	Escherichia coli APEC O1	-	+	+	-	+	-	-
iutA	Aerobactin siderophore receptor IutA	Escherichia coli APEC O1	-	+	+	-	-	-	-
iucC	aerobactin biosynthesis protein IucC	Escherichia coli APEC O1	-	+	+	-	-	-	-
iucB	aerobactin biosynthesis protein IucB	Escherichia coli APEC O1	-	+	+	-	-	-	-
iucA	aerobactin biosynthesis protein IucA	Escherichia coli APEC O1	-	+	+	-	-	-	-
shuA	TonB-dependent hemin, ferrichrome receptor	Shigella dysenteriae Sd197	+	+	+	+	+	-	-
iucD	aerobactin biosynthesis protein IucD	Shigella flexneri 2a str. 301	-	+	+	-	-	-	-
fyuA	iron aquisition outermembrane yersiniabactin receptor	Yersinia pestis CO92	-	+	+	+	+	-	-
ybtE	iron aquisition 2,3-dihydroxybenzoate-AMP ligase	Yersinia pestis CO92	-	+	+	+	+	-	-
ybtT	Iron aquisition yersiniabactin synthesis enzyme YbtT	Yersinia pestis CO92	-	+	+	+	+	-	-
ybtU	Yersiniabactin synthetase, thiazolinyl reductase component Irp3	Yersinia pestis CO92	-	+	+	+	+	-	-
irp1	iron aquisition yersiniabactin synthesis enzyme Irp1	Yersinia pestis CO92	-	+	+	+	+	-	-
irp2	iron aquisition yersiniabactin synthesis enzyme Irp2	Yersinia pestis CO92	-	+	+	+	+	-	-
ybtA	iron aquisition regulator	Yersinia pestis	-	+	+	+	+	-	-

	(YbtA,AraC-like,required for transcription of FyuA/psn,Irp2)	CO92							
ybtP	Iron siderophore ABC transporter, permease/ATP-binding protein YbtP	Yersinia pestis CO92	-	+	+	+	+	-	-
ybtQ	Iron siderophore ABC transporter, permease/ATP-binding protein YbtQ	Yersinia pestis CO92	-	+	+	+	+	-	-
ybtS	Salicylate synthetase	Yersinia pestis CO92	-	+	+	+	+	-	-
Int	Integrase	Yersinia pestis CO92	-	+	+	+	+	-	-
insD	Transposase InsD for insertion element IS2	Shigella flexneri (serotype 2a) 301	-	+	+	-	-	-	+
chuS	Hemin transport protein chuS	Escherichia coli CFT073	+	+	+	+	+	-	-
chuA	TonB-dependent hemin, ferrichrome receptor	Escherichia coli CFT073	+	+	+	+	+	-	-
chut	Periplasmic hemin-binding protein	Escherichia coli CFT073	+	+	+	+	+	-	-
chuU	Hemin ABC transporter, permease protein	Escherichia coli CFT073	+	+	+	+	+	-	-
fepA	TonB-dependent receptor	Escherichia coli CFT073	+	+	+	+	+	+	+
fepB	Ferric enterobactin-binding periplasmic protein FepB	Escherichia coli CFT073	+	+	+	+	+	+	+
fepC	Ferric enterobactin transport ATP-binding protein FepC	Escherichia coli CFT073	+	+	+	+	+	+	+
fepD	Ferric enterobactin transport system permease protein FepD	Escherichia coli CFT073	+	+	+	+	+	+	+

fepE	Ferric enterobactin uptake protein FepE	Escherichia coli CFT073	+	+	+	+	+	+	+
fepG	Ferric enterobactin transport system permease protein FepG	Escherichia coli CFT073	+	+	+	+	+	+	+
**Adhesion**									
fimB	Type 1 fimbriae regulatory protein FimB	Escherichia coli CFT073	+	+	+	+	+	+	+
fimE	Type 1 fimbriae regulatory protein FimE	Escherichia coli CFT073	+	+	+	+	+	+	+
fimA	Type-1 fimbrial protein, A chain	Escherichia coli CFT073	-	-	+	+	-	-	-
fimI	Fimbrin-like protein FimI	Escherichia coli CFT073	+	+	+	+	+	+	+
fimC	Chaperone protein FimC	Escherichia coli CFT073	+	+	+	+	+	+	+
fimD	Outer membrane usher protein FimD	Escherichia coli CFT073	+	+	+	+	+	+	+
fimF	Protein FimF (regulates length and adhesion of type 1 fimbriae)	Escherichia coli CFT073	+	+	+	+	+	+	+
fimG	Protein FimG (regulates length and adhesion of type 1 fimbriae)	Escherichia coli CFT073	+	+	+	+	+	+	+
fimH	Protein FimH (regulates length and adhesion of type 1 fimbriae, and mediates mannose binding)	Escherichia coli CFT074	+	+	+	+	+	+	+
