# Supplementary material for: Glucocorticoids Impair Phagocytosis and Inflammatory Response Against Crohn’s Disease-Associated Adherent-Invasive Escherichia coli
Source: Front Immunol. 2018 May 16;9:1026. doi: 10.3389/fimmu.2018.01026 (PMC5964128; doi:10.3389/fimmu.2018.01026)
Supplement: Supplementary file 4 [file image_1.PDF]

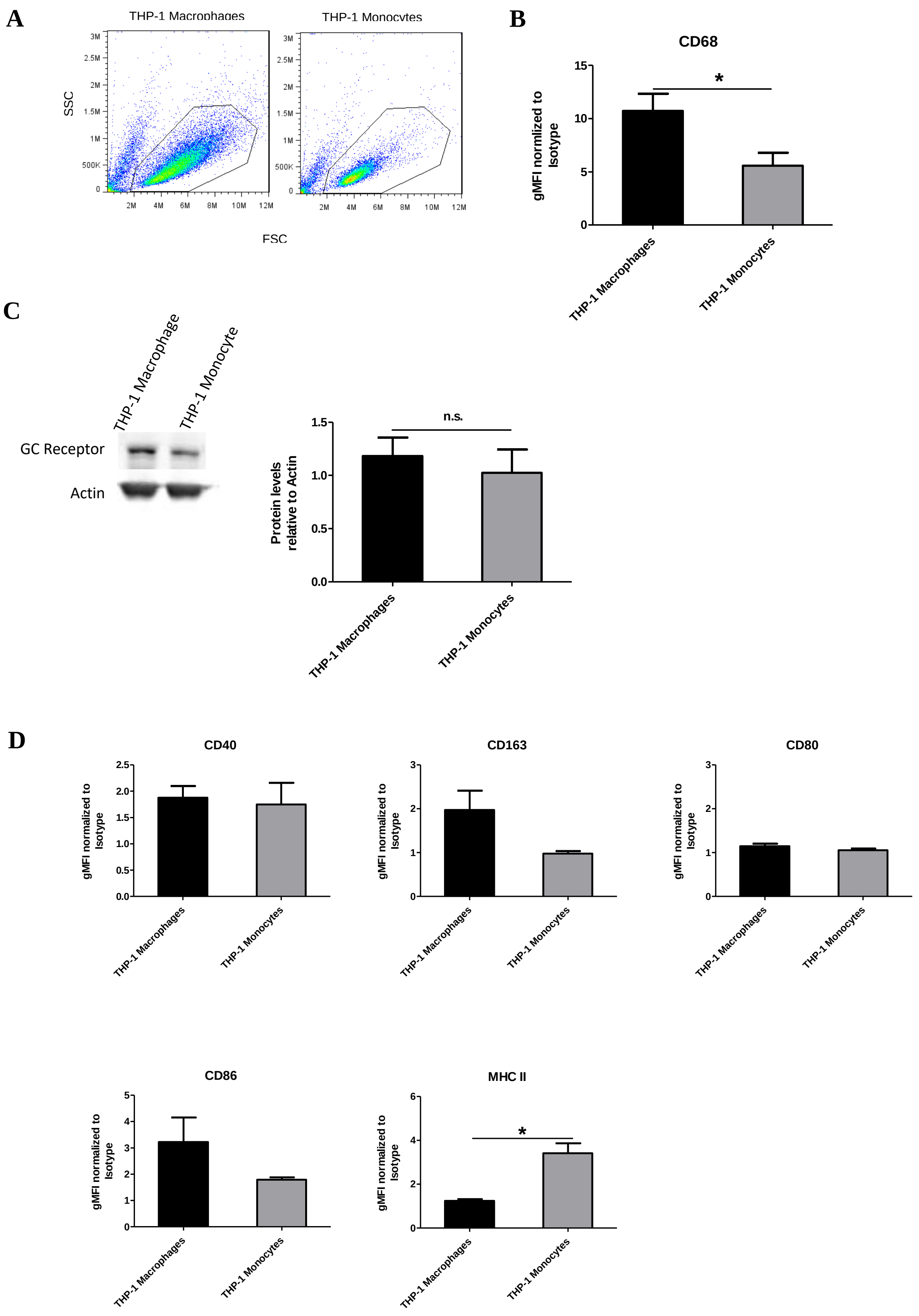
A
THP-1 Macrophages
THP-1 Monocytes
SSC
FSC
3M
2.5M
2M
1.5M
1M
500K
0
2M
4M
6M
8M
10M
12M
B
CD68
gMFI normlized to Isotype
15
10
5
0
*
THP-1 Macrophages
THP-1 Monocytes
C
THP-1 Macrophage
THP-1 Monocyte
GC Receptor
Actin
Protein levels relative to Actin
1.5
1.0
0.5
0.0
n.s.
THP-1 Macrophages
THP-1 Monocytes
D
CD40
CD163
CD80
CD86
MHC II
gMFI normalized to Isotype
2.5
2.0
1.5
1.0
0.5
0.0
3
2
1
0
5
4
6
*
THP-1 Macrophages
THP-1 Monocytes

Supplementary Figure 1. Phenotype of macrophages differentiated from THP-1 Monocyte cell line. (A) PMA differentiated macrophages are larger in size and cell complexity, as seen by flow cytometry FSC and SSC determinations, respectively. (B) Flow cytometry analysis shows that THP-1 macrophages have increased expression of the macrophage phenotype marker CD68 ($n = 3$; *= $p<0.05$). (C) Representative immunblot image of GC receptors in THP-1 macrophages and monocytes. (D) Density quantification of immunoblot images show that THP-1 macrophages maintain GR protein levels compared to THP-1 monocytes ($n = 3$; n.s.= $p>0.05$). (E) Inflammatory surface markers detected by flow cytometry show that MHC II decreases its expression after differentiation with PMA, while CD163 and CD86 increase their expression; however this distinction was not statistically significant ($n = 4$; *= $p<0.05$). Statistical comparisonas are based on two-sided Student’s t-tests.
