# Supplementary material for: Glucocorticoids Impair Phagocytosis and Inflammatory Response Against Crohn’s Disease-Associated Adherent-Invasive Escherichia coli
Source: Front Immunol. 2018 May 16;9:1026. doi: 10.3389/fimmu.2018.01026 (PMC5964128; doi:10.3389/fimmu.2018.01026)
Supplement: Supplementary file 5 [file image_2.PDF]

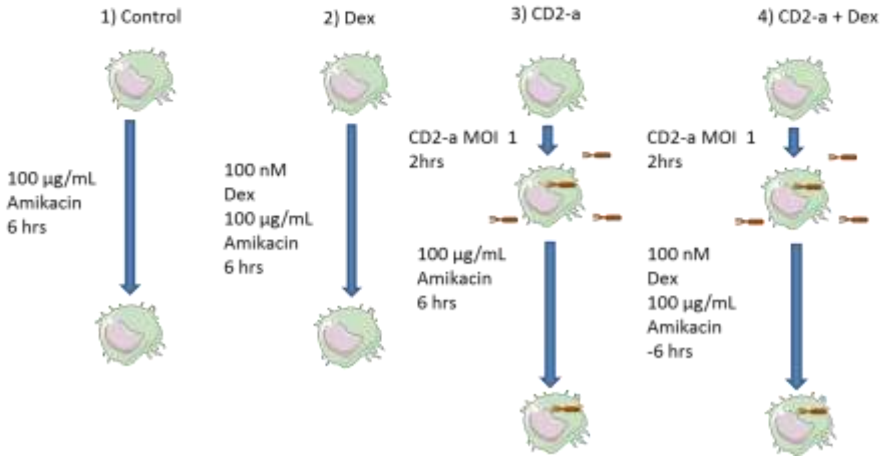

Supplementary Figure 2. Schematic repesentation of RNA microarray assay treatments. Four treatments were performed to extract RNA and execute microarray analysis: 1) Control THP-1 macrophages; 2) Dexamethasone treated THP-1 macrophages for 6 hours; 3) CD2-a treated THP-1 macrophages for 2 hours, followed by amikacin to eliminate extracellular bacteria; or 4) Dex and amikacin treated macrophages for 6 hours.
