# Supplementary material for: Glucocorticoids Impair Phagocytosis and Inflammatory Response Against Crohn’s Disease-Associated Adherent-Invasive Escherichia coli
Source: Front Immunol. 2018 May 16;9:1026. doi: 10.3389/fimmu.2018.01026 (PMC5964128; doi:10.3389/fimmu.2018.01026)
Supplement: Supplementary file 6 [file image_3.PDF]

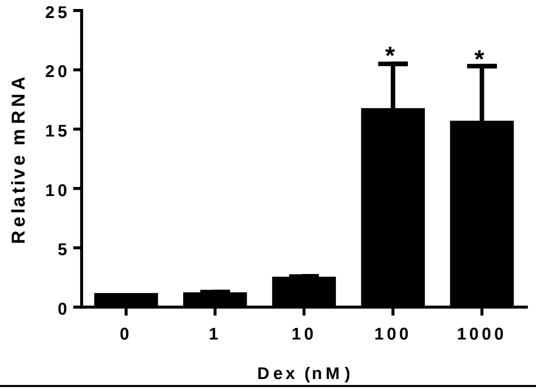

Supplementary Figure 3. Quantitative PCR determination of glucocorticoid receptor response element *GILZ*. Quantitative PCR was performed on macrophages treated with 0 - 1000 nM of Dex for 6 hours. One-way ANOVA and Bonferroni corrections were performed (n = 3; p<0.05 compared to 0).
