# Supplementary material for: Glucocorticoids Impair Phagocytosis and Inflammatory Response Against Crohn’s Disease-Associated Adherent-Invasive Escherichia coli
Source: Front Immunol. 2018 May 16;9:1026. doi: 10.3389/fimmu.2018.01026 (PMC5964128; doi:10.3389/fimmu.2018.01026)
Supplement: Supplementary file 7 [file image_4.PDF]

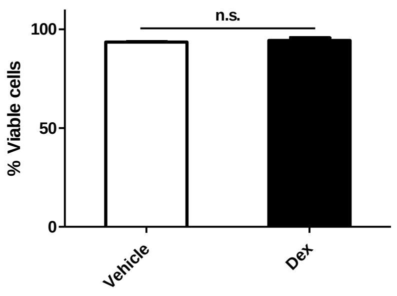

Supplementary Figure 4. Dexamethasone does not affect levels of THP-1 macrophage cell death. LDH was determined on supernatant of THP-1 macrophages treated with 100 nM of Dex or vehicle. Results shows that Dex did not modify levels of macrophage cell death after 24 hours of incubation. A two-sided Student’s t-student was perfomed (n = 3; n.s = *p*>0.05).
