# Supplementary material for: Glucocorticoids Impair Phagocytosis and Inflammatory Response Against Crohn’s Disease-Associated Adherent-Invasive Escherichia coli
Source: Front Immunol. 2018 May 16;9:1026. doi: 10.3389/fimmu.2018.01026 (PMC5964128; doi:10.3389/fimmu.2018.01026)
Supplement: Supplementary file 8 [file image_5.PDF]

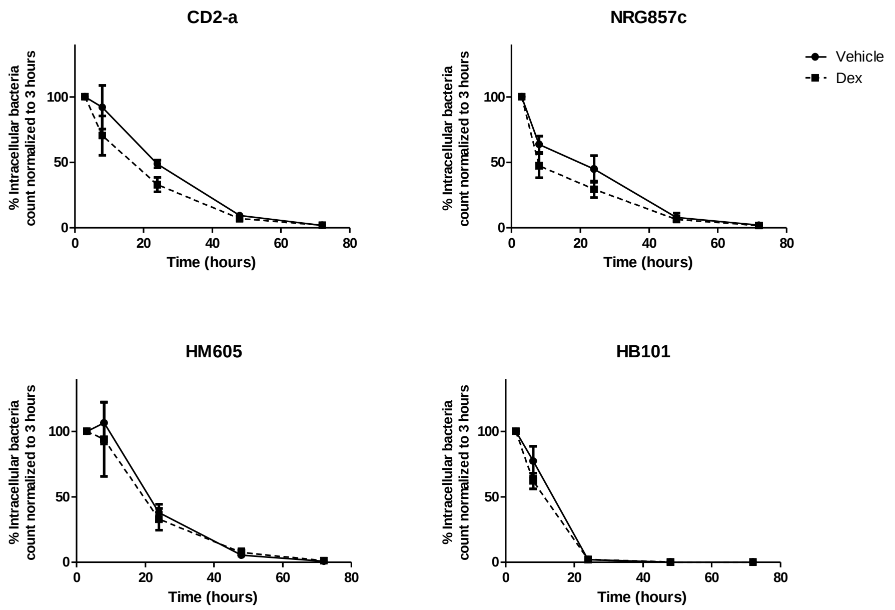

Supplementary Figure 5. Dexamethasone does not affect THP-1 macrophage bactericidal activity. Amikacin protection assay was performed from 3 to 72 hours post-infection. THP-1 macrophages were incubated with 100 nM of Dex for 24 hours prior to infection with *E. coli* strains. After 72 hours, all strains were eliminated. No statistical differences were found in the number of bacteria at any time in macrophages treated with Dex, as compared to vehicle (n =5).
