# Supplementary material for: Glucocorticoids Impair Phagocytosis and Inflammatory Response Against Crohn’s Disease-Associated Adherent-Invasive Escherichia coli
Source: Front Immunol. 2018 May 16;9:1026. doi: 10.3389/fimmu.2018.01026 (PMC5964128; doi:10.3389/fimmu.2018.01026)
Supplement: Supplementary file 9 [file image_6.PDF]

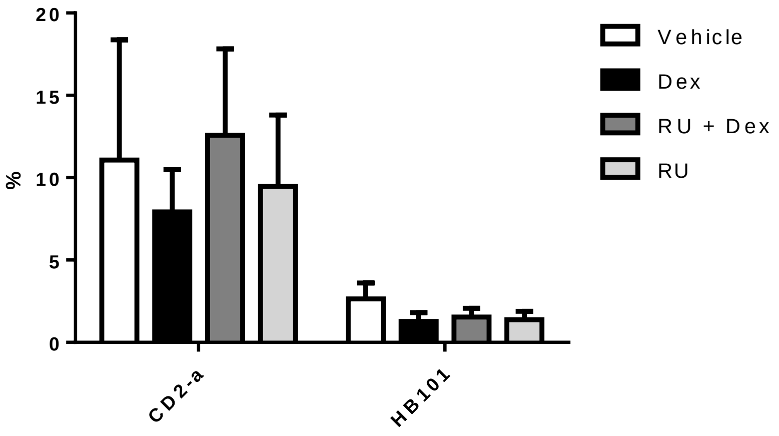

Supplementary Figure 6. Dexamethasone effect on Epithelial cell line Caco-2 suceptibility to AIEC invasion. Caco-2 cell monolayer was infected with strains HB101 and CD2a, using a multiplicity of infection (MOI) of 10 for 3 hours at 37 ° C. After washing step, cells were incubated with medium supplemented with Amikacin (100 μg/ml) for 3 more hours. Cells were lysed with Triton-X-100 at 0.1% in PBS and seeded on LC agar plates and incubated at room temperature overnight. Invasion percentage was determined as CFU/ml per hour post antibiotic treatment in relation to the initial inoculum. Two way ANOVA and Dunnet post test were performed. No statistical significance was found vs Vehicle (n = 3).
