# Supplementary material for: Glucocorticoids Impair Phagocytosis and Inflammatory Response Against Crohn’s Disease-Associated Adherent-Invasive Escherichia coli
Source: Front Immunol. 2018 May 16;9:1026. doi: 10.3389/fimmu.2018.01026 (PMC5964128; doi:10.3389/fimmu.2018.01026)
Supplement: Supplementary file 10 [file image_7.PDF]

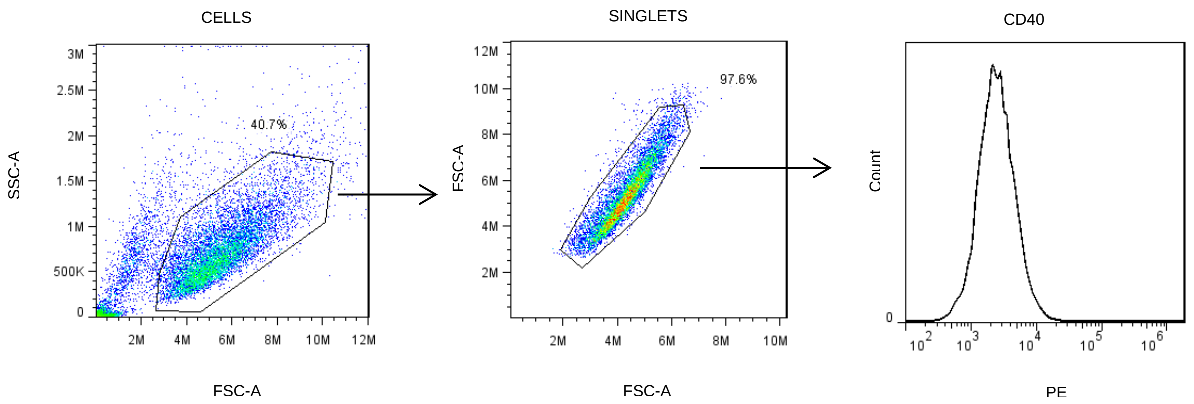

Supplementary Figure 7. Representative dot plot figure of flow cytometry gating strategy. Cells were selected by excluding debris on FSC and SSC dot plot. Next, Doublets were excluded based on FSC-A and FSC-H parameters. Geometrical mean of surface markers was calculated. This procedure was used in all Surface markers analysis (CD40, CD80, CD163, CD86, MHC class II).
