# Supplementary material for: Glucocorticoids Impair Phagocytosis and Inflammatory Response Against Crohn’s Disease-Associated Adherent-Invasive Escherichia coli
Source: Front Immunol. 2018 May 16;9:1026. doi: 10.3389/fimmu.2018.01026 (PMC5964128; doi:10.3389/fimmu.2018.01026)
Supplement: Supplementary file 11 [file image_8.PDF]

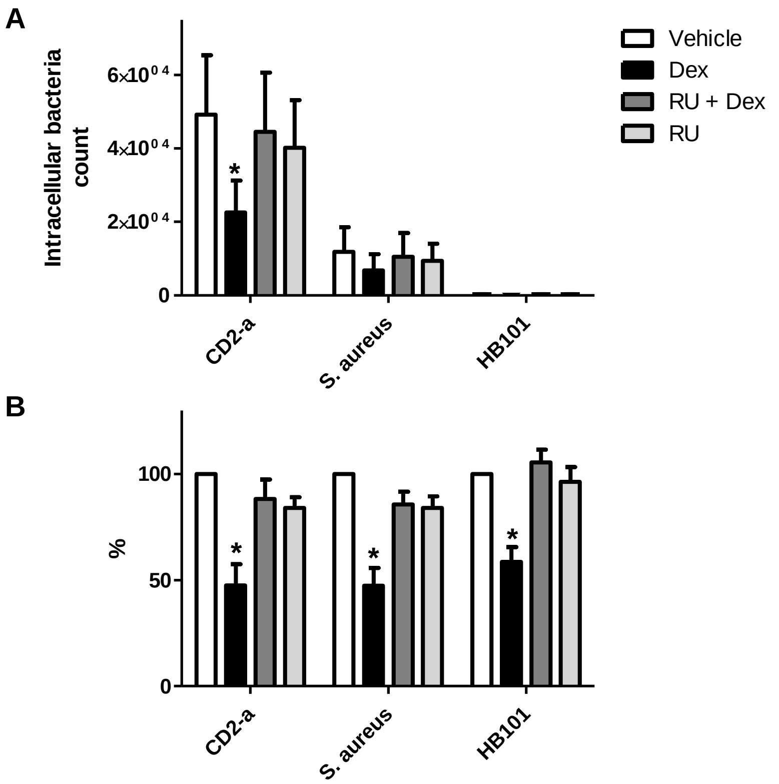

Supplementary Figure 8. Dexamethasone decreases phagocytosis of macrophages against different bacteria species. THP-1 macrophages treated for 24 hours with 100 nM Dex were infected with either *E. coli* strains CD2-a and HB101 or S. aureus (multiplicity of infecion: 10). Amikacin protection assay was performed at 30 minutes of infection to evaluate phagocytic ability. (A) Absolute intracellular CFU count, showing that different CFU of each bacteria is phagocytosed by macrophages with Dex inhibiting this process in some strains. (B) Normalization to Vehicle shows that Dex decreases phagocytosis by half in every bacteria. Two-way ANOVA and Bonferroni corrections were perfomed (n = 7; p<0.05 compared to vehicle).
